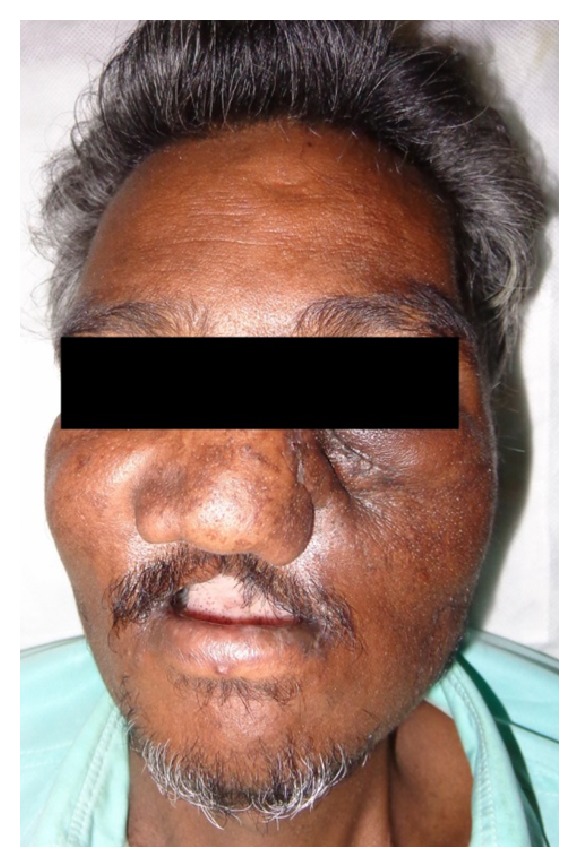# Resection and Reconstruction of Maxillary Class IIIc Defect in a Case of Adenoid Cystic Carcinoma: Cost-Sensitive Technique without Microvascular Grafts

**DOI:** 10.1155/2013/865010

**Published:** 2013-08-28

**Authors:** Dwarkadas Adwani, Anirudh Bhattacharya, Rajender Singh Arora, Ramawatar Soni, Nitin Adwani

**Affiliations:** ^1^Department of Oral & Maxillofacial Surgery, VYWS Dental College & Hospital, Amravati 444601, India; ^2^Adwani Multispeciality Dental Hospital, Ambapeth, Amravati 444601, India; ^3^Department of Head & Neck Oncology, Amravati Cancer Hospital, Amravati 444601, India; ^4^Department of Pathology, Dr. PDMMC Hospital, Amravati 444601, India

## Abstract

ACC is a rare malignant tumor that affects most commonly the major and minor salivary glands and rarely the paranasal sinuses, lacrimal gland, larynx, ear, vulva, and so forth. The maxillary sinus when affected is considered having a poor prognosis due to delayed diagnosis and delayed treatment credited to its slow spread, late symptoms, and complex anatomy which hampers surgical resection. The expressions of tumor markers too have a significant role in determining the prognosis. The treatment of choice consists of wide radical resection of the tumor followed by radiotherapy. Rehabilitation options in cases with huge maxillary defects still need further exploration.

## 1. Introduction

Adenoid cystic carcinoma (ACC) is a rare malignancy, accounting for less than 5% of all head and neck cancers [1]. ACC arises within secretory glands, most commonly the major and minor salivary glands of the head and neck. ACC can also originate from sites other than the salivary glands, such as the lacrimal gland, external ear, paranasal sinuses, larynx, tracheobronchial tree, breast, and vulva, and such ACC is called nonsalivary ACC [2]. ACC of the maxillary antrum is frequently overlooked, and therefore, patients with this tumour usually come at an advanced stage making radical resection unlikely. Difficult access and anticipated surgical morbidity are other major barriers in treatment. Biological markers Ki-67, cyclineD1, E-cadherin, and p16 also have an important impact on prognosis [3, 4]. Older age, advanced stage, positive resection margin, high histological grade, and higher expression of Ki-67 were also associated with poor outcomes.

## 2. Case Report 

**Figure 1:**
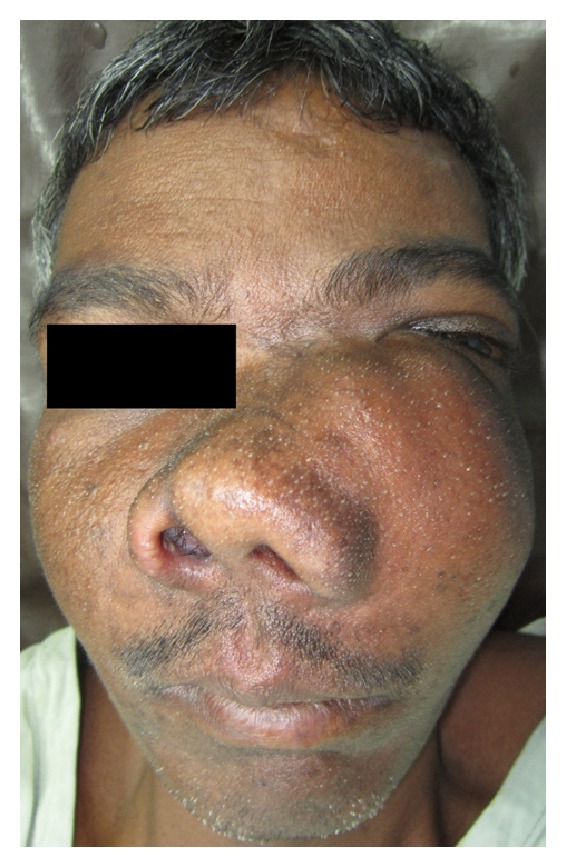


**Figure 2:**
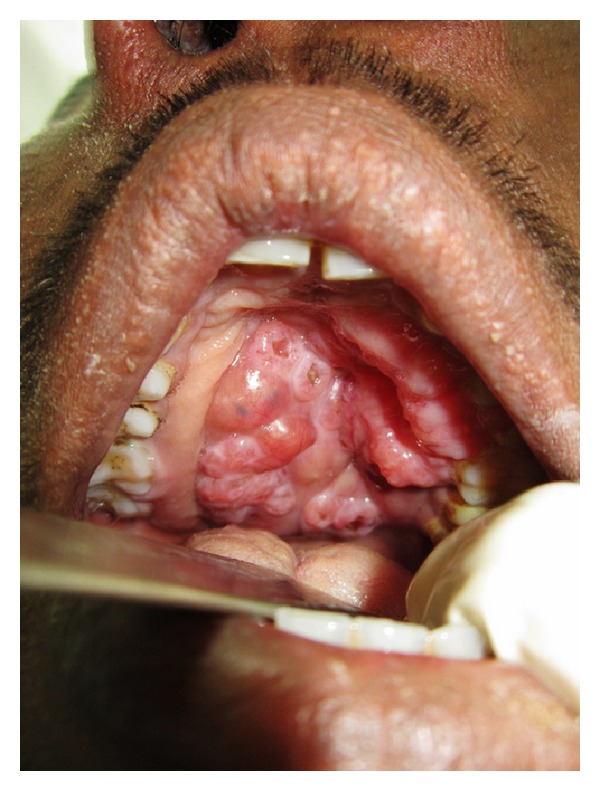


**Figure 3:**
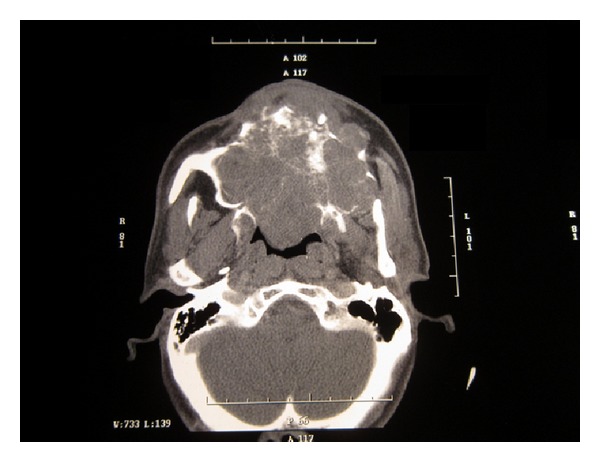


**Figure 4:**
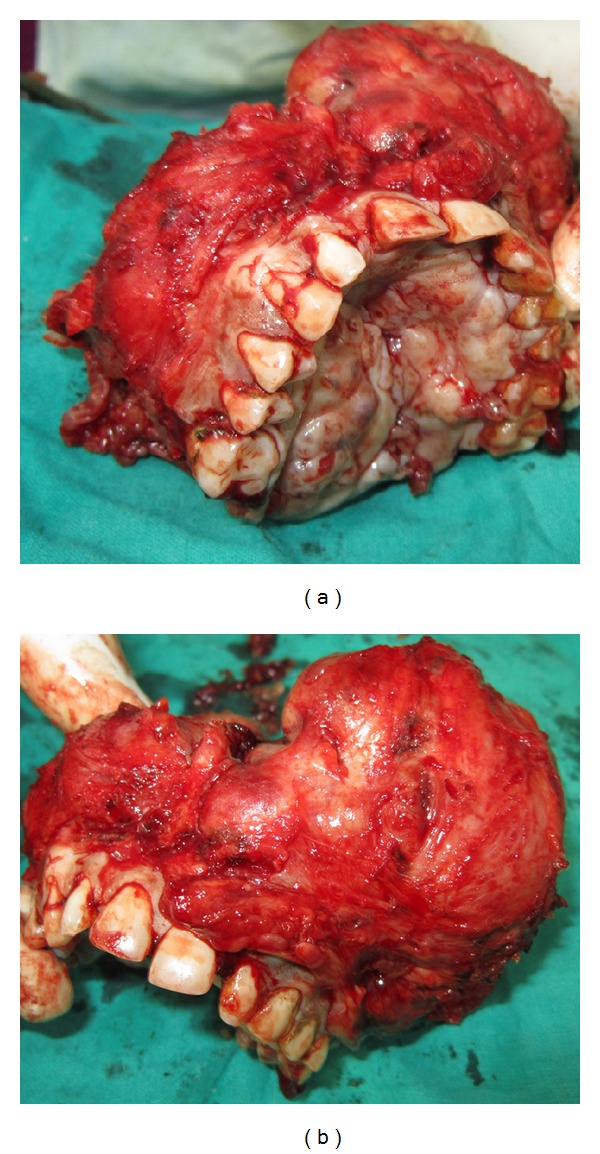


**Figure 5:**
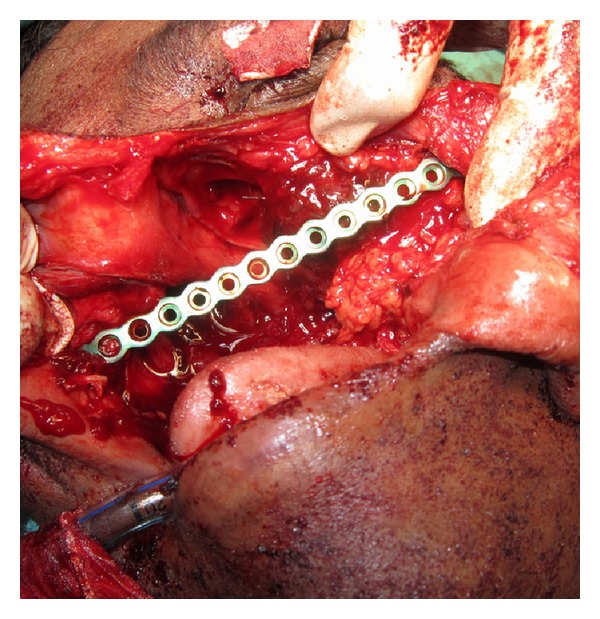


**Figure 6:**
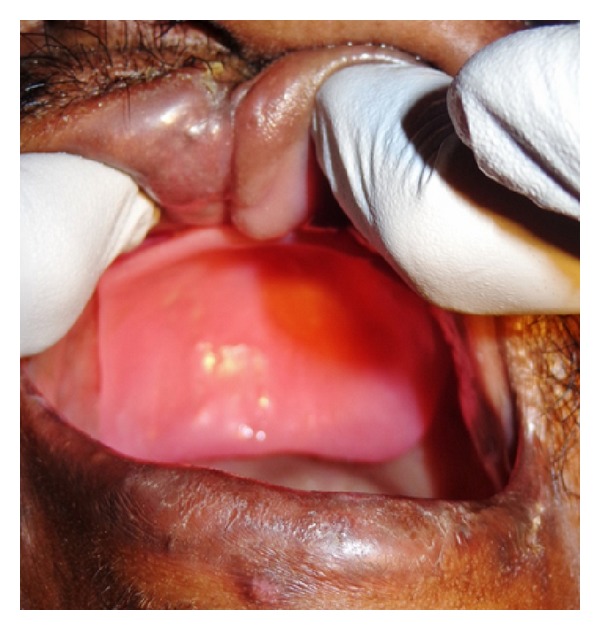


A 40-year-old male patient reported to the Department of Oral and Maxillofacial Surgery with a chief complaint of painless swelling on the left side of the face and obstruction in nasal breathing since 3 years. The swelling was slow growing, painless, and persistent in growth. There was no reduction in size of the swelling since the patient had noticed it. Personal history was negative for any detrimental habits. On extra oral examination, a large swelling was seen on left side of face, extending superoinferiorly from medial canthus of left eye till left commissure of lips and anteroposteriorly from the left lateral surface of nasal septal cartilage till 4 cm short of tragus of left ear. On nasal examination, there was severe deviation of nasal septum seen towards the right side, along with thick polyp-like mucosal obstruction in the left nostril. On eye examination, the left eye was virtually closed and raised due to the pressure from the swelling over orbit inferiorly and medially (Figure 1). Eyeball movements in all directions were normal with intact vision. Direct and consensual pupillary reflexes were present. No cervical lymphadenopathy was discernible. Intraoral examination revealed a well-defined nodular swelling covering the whole hard palate. The overlying mucosa was ulcerated on some areas and reddish in colour. It was nonfluctuant, noncompressible, and nontender. On hard tissue examination, all the permanent teeth were present, without any related dental problems (Figure 2). Laboratory blood and other serological investigations as well as ultrasonography of neck were noncontributory. Chest X-ray revealed no pleural or parenchymal abnormalities. Radiographic investigations including pantomogram, PNS Water's view, and high resolution computed tomography (HR-CT) scan were carried out. CT scan of paranasal sinuses revealed a large heterogenous mass in the left maxillary sinus, destroying all its walls crossing the midline and extending into the adjacent right nasal cavity, anterosuperiorly extending into left orbit and left ethmoidal sinus, anteroinferiorly into the hard palate and alveolar ridge, and posteroinferiorly extending into nasopharynx (Figure 3). Later, fine needle aspiration cytology was performed, and serosanguineous fluid was aspirated. Smear showed many clusters of glandular epithelial cells and eosinophilic globules along with blood cells. Findings were suggestive of a secretory gland tumor. Based on a provisional diagnosis of malignant tumor, an incisional biopsy was performed from palate near the left maxillary canine tooth. The microscopic examination of the tumour revealed features of Grade II adenoid cystic carcinoma (grading as per Szanto et al.). On immunohistochemistry examination, the tumour cells were positive for E-cadherin (Grade I, 10% of the tumour cells), positive for cyclinD1 (Grade 1+, 15% of tumour cells), showing low positivity (1%) for Ki-67, and negative for p16. Finally, based on the confirmatory diagnosis of adenoid cystic carcinoma arising from maxillary sinus, total maxillectomy along with left infraorbital rim was carried out, creating a class IIIc defect (Figures 4(a) and 4(b)). Immediate reconstruction options were very limited due to poor socioeconomic condition of the patient; therefore, we went with an unconventional method. For the loss of left infraorbital rim and floor, pedicled temporalis myofascial flap was harvested and was transpositioned into the mucosa of nasopharynx to provide an inferior base for the left globe and reduce enophthalmos. Next, to compensate for hard tissue loss, a titanium 2.5 mm continuous reconstruction plate was fixed over the body of zygoma (4 screws on either side) bilaterally to support the midfacial soft tissue structures from collapsing and threatening airway (Figure 5). The postsurgical histopathology reports confirmed the tumor-free surgical margins and the preoperative biopsy findings. Patient was sent for radiotherapy (RT), 60 gray (Gy) for a period of 45 days. After 4 weeks, a customized acrylic obturator was provided to conceal the remaining defect and to ease phonation and deglutition (Figure 6). Patient was quite comfortable with that, and his speech was pretty clear. He is in followup for the past 12 months, without any signs of recurrence or other functional difficulties except loss of masticatory ability (Figure 7). 

## 3. Discussion

Malignancies of the nasal cavity and paranasal sinuses represent only 3% to 5% of all head and neck carcinomas [5–7]. After bone invasion, the complex anatomy of the region, associated with invasion of adjacent structures and its proximity to vital structures, such as eyes, brain, and cranial nerves, has a significant direct negative impact on prognosis and survival [6, 8]. Destruction of maxillary sinus bone walls with local spreading of the tumor is common, making it difficult to reach adequate complete resection and tumor-free margins, which leads to high local recurrence rates [9]. Surgery is the treatment of choice for maxillary sinus carcinomas, and prognosis is better for patients managed by surgery followed by RT rather than for patients submitted to RT and/or chemotherapy (CT) alone [6]. Among the glandular tumors, ACC is considered to have the worst prognosis, but some authors have claimed that overall 5-year survival for maxillary sinus ACC is 57% [5, 10]. Prognosis and survival rates also depend on the histological grading of the tumour and expression of biological markers like Ki-67, cyclinD1, p16, and E-cadherin. Further, the reconstruction options in huge class IIIc defects of maxilla are limited. The conventional most favoured reconstruction modalities like free tissue transfer or the Zygoma implants are too technique sensitive and high cost bearing which cannot be executed for a part of Indian population with low socioeconomic status. In the best interest of the patient, a new cost-effective and least invasive method of supporting midfacial soft tissue structures, globe, and an obturator was tried. By this technique we were able to provide good phonation and deglutition capability to the patient, but in spite of trying our best, mastication could not be restored with such limited resources. This was the best which could be done for our patient with maxillary class IIIc defect, as in the published literature; also this type of maxillary defect is stated to be the most controversial [11]. The demand for an acceptable, very cost-effective, and least morbid technique diverted our efforts in creating something for every needy patient of such kind who cannot afford high cost and technique-sensitive microvascular tissue transfers.

## Figures and Tables

**Figure 7:**